# The Challenge of Maintaining Metabolic Health During a Global Pandemic

**DOI:** 10.1007/s40279-020-01295-8

**Published:** 2020-05-24

**Authors:** Andy J. King, Louise M. Burke, Shona L. Halson, John A. Hawley

**Affiliations:** 1 Exercise and Nutrition Research Program, The Mary Mackillop Institute for Health Research, Australian Catholic University, Level 5, 215 Spring Street, Melbourne, VIC 3000 Australia; 2 Australian Institute of Sport, Leverrier Street, Canberra, ACT 2617 Australia; 3 School of Behavioural and Health Sciences, Australian Catholic University, McAuley at Banyo, Brisbane, 4010 Australia

## Abstract

The ongoing global pandemic brought on by the spread of the novel coronavirus SARS-CoV-2 is having profound effects on human health and well-being. With no viable vaccine presently available and the virus being rapidly transmitted, governments and national health authorities have acted swiftly, recommending ‘lockdown’ policies and/or various levels of social restriction/isolation to attenuate the rate of infection. An immediate consequence of these strategies is reduced exposure to daylight, which can result in marked changes in patterns of daily living such as the timing of meals, and sleep. These disruptions to circadian biology have severe cardiometabolic health consequences for susceptible individuals. We discuss the consequences of reductions in patterns of daily physical activity and the resulting energy imbalance induced by periods of isolation, along with several home-based strategies to maintain cardiometabolic health in the forthcoming months.

## Key Points

**Table:** 

The global pandemic caused by the novel coronavirus SARS-CoV-2 may lead to deleterious health effects through reductions in daily energy expenditure, uncompensated energy intake, altered sleep, and a decrease in levels of voluntary physical activity.
Such behaviour patterns are likely to exacerbate the present public health crisis created by low levels of voluntary physical activity and the subsequent consequences for cardiometabolic health.
Some of the negative health outcomes that result from COVID-19 related isolation can be minimised by implementing graded lifestyle strategies to reduce sitting time, encourage structured physical activity, and maintain good dietary practices.

## Introduction and Background

The global pandemic caused by the severe acute respiratory syndrome coronavirus (SARS-CoV-2) has created new and unique challenges for nations throughout the world, with ramifications for individual and community health and well-being. With no viable vaccine presently available and the virus being rapidly transmitted, many governments and national health authorities have acted swiftly, recommending ‘lockdown’ policies for schools, universities, restaurants, and places of non-essential work. Various levels of restriction have also been placed on community gatherings and sporting events as well as domestic and international travel. These measures are part of the strategy by which the increase in the rate of transmission of SARS-CoV-2 and its associated illness/disease, COVID-19, can be attenuated and the virus eradicated. Persons young and old, healthy or with pre-existing medical conditions, and from diverse ethnic and socio-economic backgrounds are now faced with varying degrees of ‘isolation’ to limit potential exposure to the virus. Accordingly, it is inevitable that many individuals will be confronted with different working and living conditions, triggering personal challenges to their physical and mental health. A recent review outlined some of the psychological challenges facing individuals worldwide arising from COVID-19 enforced quarantine [1]. Here, we discuss the consequences of reductions in patterns of daily activity induced by periods of isolation along with several home-based strategies to maintain cardiometabolic health in the forthcoming months.

## Consequences of Isolation/Quarantine: Implications for Cardiometabolic Health

**Fig. 1:**
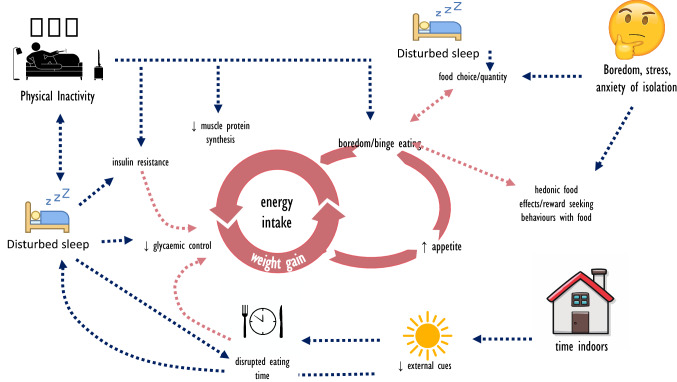
The cyclical pattern of disruptions to metabolic homeostasis during prolonged periods of COVID-19 induced isolation. A lack, or loss of physical activity, increased sitting time and changes in dietary habits and sleep lead to several physiological and psychobiological outcomes directly impacting on metabolic homeostasis. Decreased rates of skeletal muscle protein synthesis, insulin resistance and impaired immune defence have a rapid onset during periods of inactivity and are exacerbated by disruptions to sleep quality and quantity. Insulin resistance and subsequent dysregulation of glucose metabolism predispose to weight gain and increased fat mass, creating a cycle of cumulative causation in which appetite is dysregulated as the cycle continues through prolonged inactivity. External stressors and loss of sleep (quality) created by self-isolation or ‘lockdown’ scenarios can lead to alterations in food choice, timing and quantity. Self-isolation also includes reduced outdoor access and the removal or alteration of exposure to external zeitgebers such as sunlight, with flow-on effects to behaviour, sleep and metabolism

Numerous metabolic and physiological processes are underpinned by 24-h biological oscillations under the control of a central circadian clock located in the suprachiasmatic nucleus of the hypothalamus, with synchronisation of the expression of circadian clock genes primarily governed by the light–dark cycle [2]. However, epigenetic (environmental and behavioural) cues, termed ‘zeitgebers,’ can fine-tune the central clock and reset or induce time-phase shifts in circadian oscillations through suprachiasmatic nucleus-independent mechanisms. An immediate consequence of isolation/quarantine strategies is reduced exposure to daylight and the accompanying changes in patterns of physical activity, with meal timing and sleep patterns also likely to be perturbed: these ‘zeitgebers’ interact with underlying biology to create an environment in which circadian rhythms are disrupted, predisposing susceptible individuals to a plethora of metabolic abnormalities (Fig. 1).

As patterns of daily living are disturbed, a fundamental concern induced by isolation is a substantial reduction in energy expenditure. An inevitable consequence of all isolation strategies is that the majority of individuals will spend more time sitting and engaging in activities that involve very low rates of energy expenditure, such as desk-bound work, online social networking activities and television viewing. Such behaviour is likely to exacerbate the present public health crisis created by low levels of voluntary physical activity and the subsequent consequences for cardiometabolic health. Indeed, after hypertension (13%), tobacco use (9%) and prolonged hyperglycaemia (6%), physical inactivity is currently the fourth leading risk factor for global mortality, accounting for 6% of global deaths [3]. Data collected from over 30 million consumers worldwide during March 2020 by wearable technology company Fitbit indicate a substantial reduction in daily step counts compared with the corresponding period in 2019; this ranged from a 7 to 38% decline across different countries [4]. Low levels of daily physical activity and sedentary behaviour are associated with numerous adverse health outcomes including dyslipidaemia [5], microvascular dysfunction, and peripheral insulin resistance [6] that collectively predispose to weight gain (i.e., an increase in fat mass, Fig. 1) and a concomitant increase in biomarkers for cardiometabolic risk. Unfortunately, these unfavourable effects occur rapidly [6]. For example, when healthy young men decreased their daily activity levels from 10,501 to 1,344 steps/day for just two weeks, they experienced a 17% decline in skeletal muscle insulin sensitivity, a 7% decrease in cardiovascular fitness, and a 3% reduction in lean leg mass [7], explained by a reduction in myofibrillar protein synthesis rates [8]. Such metabolic perturbations are further exacerbated by periods of prolonged inactivity [9] and contribute to disruptions to whole-body homeostasis brought about by a progressive and coordinated decline in the function of numerous cells, tissues, and organs.

Dietary intake—timing, quantity and choice—is governed by a complex interaction of factors that are susceptible to change during self-isolation. Indeed various instruments which have tried to simplify the basis of dietary choice identify at least 15 different categories that underpin eating behaviour [10]. While some of these constructs are intrinsic, others will be altered during periods of self-isolation, even above that governed by inevitable changes in food availability/security at a community or household level. For example, during COVID-19 isolation we can expect large changes in social eating stimuli, emotional stimuli underpinning affect regulation and the daily routines that influence habit. What is considered ‘healthy’ might change in response to a new focus on immune support, sometimes, unfortunately, derived from non-credible sources [11]. Conversely, concepts of convenience might be altered in new daily routines, with opportunities for increased time spent on food preparation at home being associated with an increase in dietary quality [12]. Meanwhile, social media may become an even more important reference for social norms around food intake, with differential effects on different food types [13] and subgroups of the population. Self-isolation is also likely to be associated with an exacerbation of disordered eating; one expert group has reported a 30% increase in calls to an eating disorder hotline during the first week of the COVID-19 “shutdown” [14]; while, others have provided specialised information on the specific interactions between the COVID-19 virus and the presence of an eating disorder [15].

Despite heterogeneity in dietary changes during self-isolation between sub-populations and individuals, a large proportion of the community is likely to face an excess in energy intake and an increase in the time window of daily eating (i.e., the time between the first and last daily energy intake). Unless there is a correction in energy intake, significant reductions in daily energy expenditure will quickly tip the scales in favour of a positive energy balance (i.e., energy intake > energy expenditure) and subsequent weight gain. The acute relationship between appetite and physical activity is weak [16] as is the capacity for appetite to compensate for a single day of dietary overconsumption [17]. Other factors governing intake such as the hedonic effect of food [18, 19] or eating behaviour in response to boredom [20], stress [21], or anxiety [21] are likely to lead to increased energy intake secondary to changes in food choices and quantities. Currently, the average American eats over a 12-h time window [22]; while, individuals with overweight/obesity report a median daily eating duration of up to 15 h [23]. In the first longitudinal study of free-living, healthy, overweight individuals that monitored daily temporal pattern of energy intake, there was a systematic bias towards consuming a larger portion of the daily energy intake towards the late afternoon and evening hours (less than 25% of the daily caloric intake occurred before noon; while almost 40% was consumed after 18:00 h) [23]. In that study, a “three meals per day” structure of eating was largely absent, with these individuals eating throughout the wakeful hours. Fortunately, diet and exercise strategies can be implemented to increase at-home energy expenditure [24] potentially through novel, incidental exercise [25], reduce the quantity, timing and pattern of energy intake, and mitigate the potentially ‘unhealthy’ environment and behaviours associated with self-isolation.

## Lifestyle Strategies to Maintain Cardiometabolic Health During Isolation

During isolation and enforced periods of reduced energy expenditure, a primary goal for all individuals is to maintain energy balance (i.e., match energy intake to energy expenditure). This is challenging even under normal living conditions, as evident by the high prevalence of overweight/obesity in both industrialised and developing nations [26]. While confinement adds to the difficulty of conserving energy balance, several lifestyle strategies can be implemented that will have a rapid and positive impact on metabolic health in the short-term (i.e., weeks–months). In the case of severely restricted access to public gyms, swimming pools and parks, as well as restaurants, cafes and bars, we describe several home-based exercise and nutrition interventions, as well as their potential interactions with sleep, that can be implemented with minimal equipment, supervision, expense, or time commitment. We present evidence of how these ‘primary’ health drivers can help to offset the deleterious effects of inactivity and excess food intake on cardiometabolic health.

### Physical Activity

**Fig. 2:**
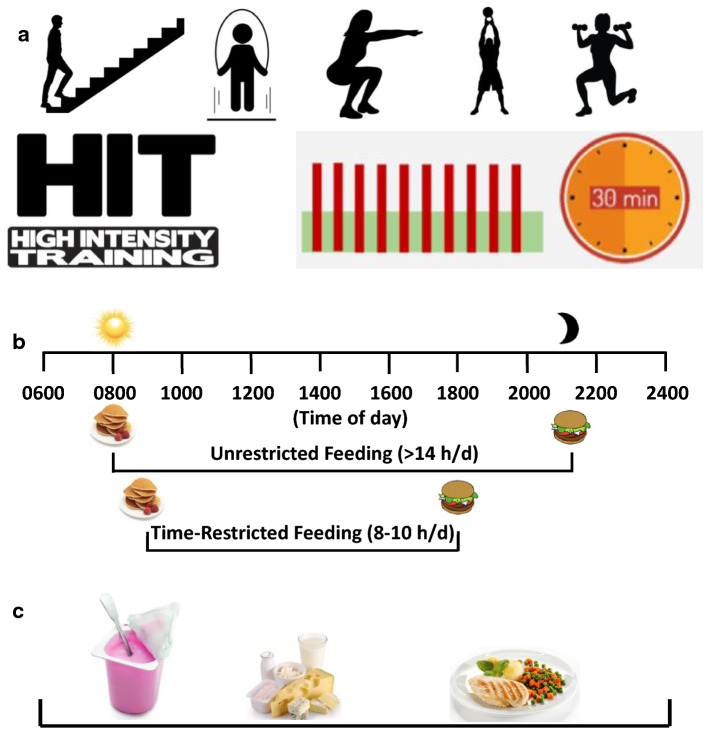
Exercise and nutrition strategies during COVID-19 isolation. A fundamental concern induced by isolation is a substantial reduction in energy expenditure. Implementing practical exercise and nutrition strategies can assist in maintaining metabolic health. **a** High-intensity interval exercise (HIT) involves short (30 s–4 min) repeated (4–10 bouts) of intense activity interspersed with 1–3 min of low- to moderate-intensity exercise or rest, and should be undertaken 3–5 times per week for 30 min. HIT rapidly induces increases in cardiorespiratory fitness as well as adaptations in skeletal muscle that assist in glycemic control. Depending on the availability of equipment, exercise prescription should be tailored to align with current/habitual levels of activity with the overall aim of reducing sedentary time (time spent sitting). “Exercise snacking” involves breaking up an exercise session into several shorter bouts spread throughout the day, and is also an effective strategy to enhance cardiorespiratory fitness compared with a single bout of the same duration. **b** Chrono-nutrition is the timing of food administration in coordination with the body’s daily rhythms, and reflects the basic idea that, in addition to the amount and content of food, the timing of meals is also critical for metabolic health. During periods of isolation, time-restricted eating (TRE), in which the daily “eating window” could be reduced from 12–14 h to 8–10 h, may help to reduce total energy intake, curtail discretionary food intake (i.e. alcohol, confectionery) in the evening, and improve overall dietary quality. **c** Consideration should be given to the timing and distribution of protein intake throughout a day to maximally stimulate rates of skeletal muscle protein synthesis. See text for details

Until recently, guidelines by major national bodies and advocacy groups recommended that physical activity should be undertaken as “continuous bouts lasting a minimum of ten minutes” to maximise cardiometabolic protection [27]. By disregarding exercise bouts lasting less than ten minutes, these guidelines ignored potential health benefits induced by briefer bouts of high-intensity activities, and in 2018, US and UK physical activity guidelines explicitly removed this 10-min ‘minimum bout’ requirement [28, 29]. An established body of scientific evidence and widespread public interest in the potential for high-intensity intermittent exercise (HIT) to provoke physiological adaptations that are similar, or even superior, to traditional endurance exercise training in healthy individuals, as well as those with lifestyle-induced cardiometabolic disorders, was instrumental in bringing about this change [30]. HIT is infinitely variable, but can be defined as short (30 s–4 min) repeated (4–10 bouts) of intense activity interspersed with 1–3 min of low- to moderate-intensity exercise or rest. There are rapid improvements in markers of metabolic health in healthy but previously untrained individuals, who demonstrate marked increases in cardiovascular fitness, skeletal muscle mitochondrial density and insulin sensitivity after just two weeks of HIT (three sessions per week, a total of ~ 15 min of intense exercise) [31]. HIT may also reduce appetite in the immediate hours following a single session of training [32], making the timing of exercise during the day an important consideration. While laboratory-based studies of HIT interventions have typically used cycle ergometry or treadmill running as modes of exercise, it is unlikely most households will have access to such equipment. In this regard, recent studies support the use of various protocols of brief intense stair-climbing [33] and whole-body exercise for improving cardiorespiratory fitness and conferring many of the metabolic and physiological adaptations induced by laboratory-based protocols (see Fig. 2).

High-intensity interval training may, however, be unsuitable for individuals with pre-existing cardiometabolic disease, or a predominantly sedentary lifestyle. As such, graded strategies to increase physical activity are advised, with the first step a reduction in sitting time through light ambulation. “Exercise snacking” which involves breaking up an exercise session into several shorter bouts spread throughout the day is also an effective strategy to enhance cardiorespiratory fitness compared with a single bout of the same duration [34]. In those who are overweight and insulin resistant, dosing exercise as brief, intense 'exercise snacks' before main meals is an effective approach to improve glycaemic control [35]. While implementation of a daily exercise regimen will increase energy expenditure, simply reducing sitting time attenuates the decline in cardiometabolic health, independent of any structured physical activity [36]. Individuals should, therefore, focus on limiting periods of prolonged inactivity and undertaking planned exercise/movement snacks. Where permitted, outdoor exercise should be continued, while adhering to “social distancing” regulations.

Maintaining skeletal muscle mass is difficult in the face of reduced levels of habitual activity [7] and especially difficult for the elderly and those with a physical disability. Resistance-type exercise (overloading muscle to a greater extent than can be attained during daily living activities) stimulates the synthesis of skeletal muscle protein, ultimately resulting in skeletal muscle hypertrophy [37]. A common belief is that the greatest gains in strength and muscle mass are achieved when the loading is high (i.e., when the resistance is close to maximal). However, recent data demonstrate that low-load (30% of one repetition maximum, 1RM) high-volume (until volitional failure) resistance-type exercise has potent and stimulatory effects on an array of anabolic signalling molecules and results in similar increases in the rates of muscle protein synthesis (MPS) as high loads (90% 1RM to failure) [38]. Accordingly, for previously untrained and elderly individuals, home-based resistive exercise using bodyweight or microbands may provide sufficient overload for maintaining lean mass. As dietary protein supplementation significantly enhances muscle strength and size during resistance-based training in healthy adults [39] and attenuates potential inactivity-induced loss of muscle protein [40], daily protein intake during prolonged periods of reduced activity and isolation should be increased to 1.2 g/kg body mass/day [39]. As the timing and distribution of protein ingestion is a key factor in maximally stimulating rates of MPS throughout an entire day [41], 20–25 g of protein should be ingested soon after an exercise bout with similar amounts consumed at regular 3–4-h intervals throughout the day. However, individuals must remain cognisant of total energy intake throughout the day, and be aware of reduced energy requirements in isolation by making adjustments to other macronutrients in meals and snacks.

### Chrono-Nutrition

The timing of meals affects a wide variety of physiological functions, including the sleep/wake cycle, core body temperature, athletic performance, and mental alertness [42, 43]. Furthermore, the timing of meals has a profound effect on skeletal muscle insulin sensitivity and whole-body metabolic health: manipulation of the feeding-fasting cycle and reducing the time spent in a postprandial and postabsorptive state improve glycaemic control, while perturbing feeding-fasting cycles drives robust oscillations in metabolism and circadian rhythms that maintain cardiometabolic health [44]. In humans, insulin sensitivity, β-cell responsiveness, and the thermic effect of food are all higher in the morning than in the afternoon or evening [43], suggesting that human metabolism is geared towards a greater food intake in the morning than the evening. Indeed, changes in food composition/feeding time lead to differential activation of epigenetic and transcriptional control systems through harnessing specialised enzymatic pathways and circadian metabolic sensors [42]. The results of human studies reveal that eating in alignment with circadian rhythms (increasing food intake at breakfast and reducing it at dinner time) improves glycaemic control, weight loss and lipid levels [44, 45] without increasing hunger [23]. In contrast, irregular daily eating patterns have adverse effects on circadian biology [46] independent of meal size and macronutrient composition [47]. As such, the concept of “chrono-nutrition” refers to food administration in coordination with the body’s daily rhythms, and reflects the basic idea that, in addition to the amount and content of food, the timing of meals is also critical for metabolic health [42].

Excess energy intake caused by overeating, particularly ‘discretionary’ or ‘comfort’ foods late at night is likely to be a short-term outcome of isolation. Dieting, including the exclusion of individual foods and/or food groups (i.e., ketogenic, vegan, paleo diets), has low compliance and adherence for the majority of the population. Furthermore, the belief that it is important to eat three or more meals per day on a regular basis is deeply ingrained in most societies; so changes to this pattern will likely be met by resistance. As such there is need to implement home-based dietary approaches that are socially acceptable, feasible, and achievable over the short- to medium term. While permutations in the pattern of daily food consumption are numerous, one such practical strategy to maintain cardiometabolic health during periods of self-isolation is time-restricted eating (TRE), in which the normal daily eating duration is reduced from a 12–14 h to a 10 h/day “eating window” (see Fig. 2). Several protocols of TRE have been studied in humans, with positive outcomes on a plethora of health markers. Sutton et al. [44] reported that men with prediabetes who completed five weeks of a strict regimen of TRE (a 6 h TRE period, with dinner consumed before 15:00 h) improved insulin sensitivity and β-cell responsiveness and reduced blood pressure and markers of oxidative stress compared to a 12-h feeding protocol. In that study, participants lived in a metabolic ward for the duration of the investigation, and meals provided to participants were matched to energy requirements, so they did not lose weight. Remarkably, the observed improvements in cardiometabolic health were independent of weight loss. However, it is highly unlikely that individuals faced with periods of self-isolation would ever choose to follow such a strict eating regimen.

Recently, Parr et al. [45] determined the effects of a modified form of TRE (8 h/day, meals consumed at 10:00, 13:00 and 17:00 h) versus extended feeding (15 h/day, consuming meals at 0700, 1400 and 2100 h) on 24-h and postprandial metabolism in men with overweight/obesity. TRE improved nocturnal and postprandial glycaemic control and this protocol was well accepted by the overweight/obese males in that study. It appears that TRE offers a practical advantage over stricter energy-restricted diet interventions for individuals in self-isolation, given there are no rigid requirements around energy restriction or discretionary food choices. As noted, the types of foods we consume often are aligned closely to distinct times of the day; alcohol typically is consumed at the end of the day, as are sweet (refined sugar) foods such as ice cream [23]. A reduction in food intake later in the day may not only reduce total energy intake but also curtail discretionary food intake and improve overall dietary quality. However, it also is possible that placing time restrictions on eating could result in poorer food choices in some individuals.

### Sleep

Significant lifestyle alterations associated with home confinement may result in changes in sleep quality, quantity and timing [48]. As noted, circadian rhythm is influenced primarily by the light/dark cycle, and ‘fine tuned’ by the timing of meals and levels of activity/inactivity, all of which exert a profound influence on the sleep/wake cycle. Light synchronises the circadian rhythm to the external environment and disruption to the external light/dark cycle can have a negative impact on sleep. Individuals who are self-isolating may be exposed to less daylight than usual which may lead to increased sleep disturbances [48].

**Fig. 3:**
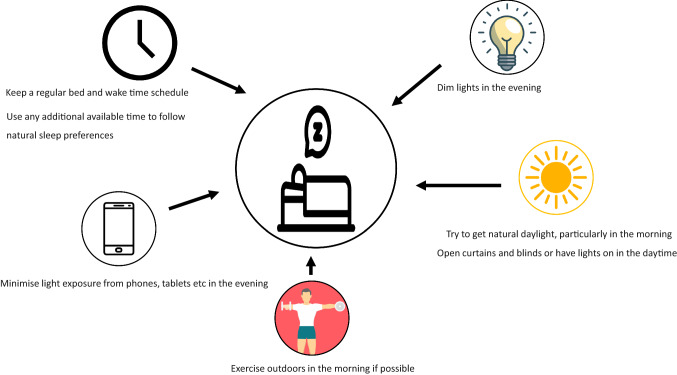
Summary of practical sleep strategies during isolation. The goal is to optimise sleep quality, duration and consistency. A regular daily sleep schedule, avoiding unnecessary naps (unless sleep deprived), exposure to sunlight in the morning, avoiding bright lights (including electronic devices) in the evening, and following natural sleep preference will help to achieve this goal

Exercise is recognised as a safe, inexpensive and accessible means of improving sleep and has been proposed as an alternative treatment for insomnia [49]. The potential mechanisms for this improvement are likely to include the influence of light exposure on circadian rhythms, an increase in energy expenditure and body temperature (increasing requirement for recovery) and anti-anxiolytic and anti-depressant effects of exercise [49]. In comparison to exercise, much less is known about the potential negative influence of sedentary behaviour on sleep. However, a recent meta-analysis concluded that sedentary behaviour was associated with an increased risk of insomnia and sleep disturbance [50]. It is possible that time spent in sedentary ‘distractions’ such as television watching and phone and computer use may take the place of sleep and result in shorter sleep durations, and, in particular, may disturb melatonin release due to light exposure from these devices (Fig. 3).

There is emerging interest in the role of intraindividual variability in sleep and wake times and the impact it may have on not only sleep quality, but also metabolic health [51, 52] and associated mental and physical outcomes [53]. Many individuals who are isolating may find that due to a loss of commute/travel time and a decrease in social activities, there is actually more time to sleep, especially at times which were not previously possible (i.e. the ability to ‘sleep in’ or nap). The interaction of diet and meal timing with sleep is a relatively new area of research and likely influences sleep quality and quantity. Evidence suggests that some nutritional interventions may impact sleep [54] and that alignment of sleep and meal times may influence food choices and energy balance in a bi-directional manner [55]. Finally, given the associations between poor sleep and obesity, insulin resistance and reduced glycemic control [56, 57], optimising sleep is likely to play a very significant role in the maintenance of metabolic health.

## Conclusion

The ongoing global crisis brought on by the spread of the COVID-19 virus will have profound effects on human health and well-being. In situations in which large numbers of the community are required to isolate and ‘lockdown’ measures imposed, deleterious health effects will be likely due to a reduction in daily energy expenditure and an increase, or failure to appropriately reduce, energy intake. Individuals can attenuate some of the negative health outcomes that result from isolation by implementing practical lifestyle strategies that encourage energy balance. Exercise, nutrition, and sleep play a fundamental role in human health and physiology. Several metabolic processes and pathways converge on key indices of physiological health and are significantly impacted by poor sleep, physical inactivity, time indoors and the potential anxiety created by isolation (Fig. 1). While structured physical activity alleviates the consequence of positive energy balance, simply reducing total sitting time is a vital first step to maintaining cardiometabolic health. Additionally, good dietary practice can mitigate metabolic disturbances, and awareness of adequate protein intake and meal timing, while ensuring energy intake does not suffer from isolation-related food intake and inactivity, is essential. Physical activity and good dietary practice have a bidirectional influence on sleep, and together play a fundamental role in not only cardiometabolic health, but may also be protective factors to cope positively with isolation-related challenges.

## References

[1] Brooks SK, Webster RK, Smith LE, Woodland L, Wessely S, Greenberg N (2020). The psychological impact of quarantine and how to reduce it: Rapid review of the evidence. Lancet.

[2] Eckel-Mahan K, Sassone-Corsi P (2013). Metabolism and the circadian clock converge. Physiol Rev.

[3] Organization WH. Global recommendations on physical activity for health. Geneva2010. 26180873

[4] Fitbit. The impact of coronavirus on global activity. 2020. https://blog.fitbit.com/covid-19-global-activity/. Accessed 29 Mar 2020.

[5] Homer AR, Owen N, Dunstan DW (2019). Too much sitting and dysglycemia: Mechanistic links and implications for obesity. Curr Opin Endocr Metab Res.

[6] Hamburg NM, McMackin CJ, Huang AL, Shenouda SM, Widlansky ME, Schulz E (2007). Physical inactivity rapidly induces insulin resistance and microvascular dysfunction in healthy volunteers. Arterioscler Thromb Vasc Biol.

[7] Krogh-Madsen R, Thyfault JP, Broholm C, Mortensen OH, Olsen RH, Mounier R, et al. A 2-wk reduction of ambulatory activity attenuates peripheral insulin sensitivity. J Appl Physiol (1985). 2010;108(5):1034–40. 10.1152/japplphysiol.00977.2009. 10.1152/japplphysiol.00977.2009 20044474

[8] Shad BJ, Thompson JL, Holwerda AM, Stocks B, Elhassan YS, Philp A (2019). One week of step reduction lowers myofibrillar protein synthesis rates in young men. Med Sci Sports Exerc.

[9] Hamilton MT, Hamilton DG, Zderic TW (2007). Role of low energy expenditure and sitting in obesity, metabolic syndrome, type 2 diabetes, and cardiovascular disease. Diabetes.

[10] Renner B, Sproesser G, Strohbach S, Schupp HT. Why we eat what we eat. The eating motivation survey (tems). Appetite. 2012;59(1):117–28. https:doi.org/10.1016/j.appet.2012.04.004. 10.1016/j.appet.2012.04.004 22521515

[11] Marr B. Coronavirus fake news: how facebook, twitter, and instagram are tackling the problem. Jersey City, New Jersey, U.S. 2020. https://www.forbes.com/sites/bernardmarr/2020/03/27/finding-the-truth-about-covid-19-how-facebook-twitter-and-instagram-are-tackling-fake-news/#4ea493321977. Accessed 27 Mar 2020.

[12] Monsivais P, Aggarwal A, Drewnowski A (2014). Time spent on home food preparation and indicators of healthy eating. Am J Prev Med.

[13] Hawkins LK, Farrow C, Thomas JM (2020). Do perceived norms of social media users' eating habits and preferences predict our own food consumption and bmi?. Appetite.

[14] Gallagher S (2020). Coronavirus: eating disorder helpline has seen calls rise 30% during outbreak. Independent.

[15] Muhlheim L. Eating disorders during the coronavirus (covid-19) pandemic. Verywellmind.com, New York, New York. 2020. https://www.verywellmind.com/eating-disorders-during-the-covid-19-pandemic-4800648. Accessed 25 Mar 2020.

[16] Stensel DJ, King JA, Thackray AE (2016). Role of physical activity in regulating appetite and body fat. Nutr Bull.

[17] Deighton K, King AJ, Matu J, Shannon OM, Whiteman O, Long A (2019). A single day of mixed-macronutrient overfeeding does not elicit compensatory appetite or energy intake responses but exaggerates postprandial lipaemia during the next day in healthy young men. Br J Nutr.

[18] Coccurello R, Maccarrone M (2018). Hedonic eating and the "delicious circle": from lipid-derived mediators to brain dopamine and back. Front Neurosci.

[19] Hopkins M, Gibbons C, Caudwell P, Blundell JE, Finlayson G (2016). Differing effects of high-fat or high-carbohydrate meals on food hedonics in overweight and obese individuals. Br J Nutr.

[20] Moynihan AB, van Tilburg WA, Igou ER, Wisman A, Donnelly AE, Mulcaire JB (2015). Eaten up by boredom: consuming food to escape awareness of the bored self. Front Psychol.

[21] Yau YHC, Potenza MN (2013). Stress and eating behaviors. Miner Endocrinol.

[22] Kant AK, Graubard BI (2014). Association of self-reported sleep duration with eating behaviors of american adults: Nhanes 2005–2010. Am J Clin Nutr.

[23] Gill S, Panda S (2015). A smartphone app reveals erratic diurnal eating patterns in humans that can be modulated for health benefits. Cell Metab.

[24] Gibala MJ, Little JP (2020). Physiological basis of brief vigorous exercise to improve health. J Physiol.

[25] Stamatakis E, Johnson NA, Powell L, Hamer M, Rangul V, Holterman A (2019). Short and sporadic bouts in the 2018 us physical activity guidelines- is high-intensity incidental physical activity the new hiit?. Br J Sports Med.

[26] Abarca-Gómez L, Abdeen ZA, Hamid ZA, Abu-Rmeileh NM, Acosta-Cazares B, Acuin C (2017). Worldwide trends in body-mass index, underweight, overweight, and obesity from 1975 to 2016: A pooled analysis of 2416 population-based measurement studies in 128·9 million children, adolescents, and adults. Lancet.

[27] Haskell WL, Lee IM, Pate RR, Powell KE, Blair SN, Franklin BA (2007). Physical activity and public health: updated recommendation for adults from the american college of sports medicine and the american heart association. Med Sci Sports Exerc.

[28] Piercy KL, Troiano RP, Ballard RM, Carlson SA, Fulton JE, Galuska DA (2018). The physical activity guidelines for americans. JAMA.

[29] Department of Health and Social Care. Uk chief medical officers' physical activity guidelines of Work. United Kingdom. 2019.

[30] Gibala MJ, Little JP, Macdonald MJ, Hawley JA (2012). Physiological adaptations to low-volume, high-intensity interval training in health and disease. J Physiol.

[31] MacInnis MJ, Gibala MJ (2017). Physiological adaptations to interval training and the role of exercise intensity. J Physiol.

[32] Islam H, Townsend LK, McKie GL, Medeiros PJ, Gurd BJ, Hazell TJ. Potential involvement of lactate and interleukin-6 in the appetite-regulatory hormonal response to an acute exercise bout. J Appl Physiol (1985). 2017;123(3):614–23. 10.1152/japplphysiol.00218.2017. 10.1152/japplphysiol.00218.2017 PMC5625078 28684587

[33] Jenkins EM, Nairn LN, Skelly LE, Little JP, Gibala MJ (2019). Do stair climbing exercise "snacks" improve cardiorespiratory fitness?. Appl Physiol Nutr Metab.

[34] Murphy MH, Lahart I, Carlin A, Murtagh E (2019). The effects of continuous compared to accumulated exercise on health: a meta-analytic review. Sports Med.

[35] Francois ME, Baldi JC, Manning PJ, Lucas SJ, Hawley JA, Williams MJ (2014). 'Exercise snacks' before meals: a novel strategy to improve glycaemic control in individuals with insulin resistance. Diabetologia.

[36] Bankoski A, Harris TB, McClain JJ, Brychta RJ, Caserotti P, Chen KY (2011). Sedentary activity associated with metabolic syndrome independent of physical activity. Diabetes Care.

[37] McGlory C, van Vliet S, Stokes T, Mittendorfer B, Phillips SM (2019). The impact of exercise and nutrition on the regulation of skeletal muscle mass. J Physiol.

[38] Burd NA, West DW, Staples AW, Atherton PJ, Baker JM, Moore DR (2010). Low-load high volume resistance exercise stimulates muscle protein synthesis more than high-load low volume resistance exercise in young men. PLoS ONE.

[39] Morton RW, Murphy KT, McKellar SR, Schoenfeld BJ, Henselmans M, Helms E (2018). A systematic review, meta-analysis and meta-regression of the effect of protein supplementation on resistance training-induced gains in muscle mass and strength in healthy adults. Br J Sports Med.

[40] Galvan E, Arentson-Lantz E, Lamon S, Paddon-Jones D. Protecting skeletal muscle with protein and amino acid during periods of disuse. Nutrients. 2016;8(7). 10.3390/nu8070404. 10.3390/nu8070404 PMC4963880 27376322

[41] Areta JL, Burke LM, Ross ML, Camera DM, West DW, Broad EM (2013). Timing and distribution of protein ingestion during prolonged recovery from resistance exercise alters myofibrillar protein synthesis. J Physiol.

[42] Asher G, Sassone-Corsi P (2015). Time for food: the intimate interplay between nutrition, metabolism, and the circadian clock. Cell.

[43] Parr EB, Heilbronn LK, Hawley JA (2020). A time to eat and a time to exercise. Exerc Sport Sci Rev.

[44] Sutton EF, Beyl R, Early KS, Cefalu WT, Ravussin E, Peterson CM. Early time-restricted feeding improves insulin sensitivity, blood pressure, and oxidative stress even without weight loss in men with prediabetes. Cell Metab. 2018;27(6):1212–21 e3. 10.1016/j.cmet.2018.04.010. 10.1016/j.cmet.2018.04.010 PMC5990470 29754952

[45] Parr EB, Devlin BL, Radford BE, Hawley JA. A delayed morning and earlier evening time-restricted feeding protocol for improving glycemic control and dietary adherence in men with overweight/obesity: a randomized controlled trial. Nutrients. 2020;12(2). 10.3390/nu12020505. 10.3390/nu12020505 PMC7071240 32079327

[46] Delezie J, Challet E (2011). Interactions between metabolism and circadian clocks: reciprocal disturbances. Ann N Y Acad Sci.

[47] Hatori M, Vollmers C, Zarrinpar A, DiTacchio L, Bushong EA, Gill S (2012). Time-restricted feeding without reducing caloric intake prevents metabolic diseases in mice fed a high-fat diet. Cell Metab.

[48] Altena E, Baglioni C, Espie CA, Ellis J, Gavriloff D, Holzinger B, et al. Dealing with sleep problems during home confinement due to the covid-19 outbreak: practical recommendations from a task force of the european cbt-i academy. J Sleep Res. 2020:e13052. https:doi.org/10.1111/jsr.13052. 10.1111/jsr.13052 32246787

[49] Lowe H, Haddock G, Mulligan LD, Gregg L, Fuzellier-Hart A, Carter LA (2019). Does exercise improve sleep for adults with insomnia? A systematic review with quality appraisal. Clin Psychol Rev.

[50] Yang Y, Shin JC, Li D, An R (2017). Sedentary behavior and sleep problems: A systematic review and meta-analysis. Int J Behav Med.

[51] Caia J, Halson SL, Scott TJ, Kelly VG (2017). Intra-individual variability in the sleep of senior and junior rugby league athletes during the competitive season. Chronobiol Int.

[52] Bei B, Manber R, Allen NB, Trinder J, Wiley JF. Too long, too short, or too variable? Sleep intraindividual variability and its associations with perceived sleep quality and mood in adolescents during naturalistically unconstrained sleep. Sleep. 2017;40(2). 10.1093/sleep/zsw067. 10.1093/sleep/zsw067 28364491

[53] Bei B, Wiley JF, Trinder J, Manber R (2016). Beyond the mean: a systematic review on the correlates of daily intraindividual variability of sleep/wake patterns. Sleep Med Rev.

[54] Halson SL (2014). Sleep in elite athletes and nutritional interventions to enhance sleep. Sports Med.

[55] St-Onge MP, Pizinger T, Kovtun K, RoyChoudhury A (2019). Sleep and meal timing influence food intake and its hormonal regulation in healthy adults with overweight/obesity. Eur J Clin Nutr.

[56] Reutrakul S, Van Cauter E (2018). Sleep influences on obesity, insulin resistance, and risk of type 2 diabetes. Metabolism.

[57] Lee SWH, Ng KY, Chin WK (2017). The impact of sleep amount and sleep quality on glycemic control in type 2 diabetes: a systematic review and meta-analysis. Sleep Med Rev.

